# Corrective Osteotomy with Volar and Dorsal Fixation for Malunion of Intra‐Articular Fracture of the Distal Radius: A Retrospective Study

**DOI:** 10.1111/os.13409

**Published:** 2022-07-22

**Authors:** Huihao Zhang, Yong Zhu, Fangda Fu, Lingyun Gou, Yonglin Zhu, Zhiguo Zhang, Chengcong Zhou, Sai Yao, Ming Yue, Xiaofeng Li, Peijian Tong, Hongfeng Ruan, Chengliang Wu

**Affiliations:** ^1^ Institute of Orthopaedics and Traumatology The First Affiliated Hospital of Zhejiang Chinese Medical University Hangzhou China; ^2^ Department of Orthopaedic Surgery Liuzhou Traditional Chinese Medical Hospital Liuzhou China; ^3^ Department of Physiology Zhejiang Chinese Medical University Hangzhou China; ^4^ Department of Orthopedics and Traumatology, Shanghai Municipal Hospital of Traditional Chinese Medicine Shanghai University of Traditional Chinese Medicine Shanghai China

**Keywords:** Corrective osteotomy, Distal radius fracture, Dorsal fixation, Malunion, Volar fixation

## Abstract

**Objectives:**

Although corrective osteotomy with volar or dorsal plate fixation can treat malunion of distal radius fractures, each has its own disadvantages. Little is currently known on whether dorsal fixation combined with volar fixation may further improve recovery. This study aimed to evaluate the clinical value of corrective osteotomy combined with volar and dorsal plate fixation in patients with malunion of intra‐articular fractures of the distal radius.

**Methods:**

Seventeen patients with malunion of intra‐articular fractures of the distal radius treated with corrective osteotomy with volar and dorsal plate fixation from 1 January 2016 to 31 November 2018 were retrospectively analyzed. The enrolled patients included seven males and 10 females with an average age of 54.9 years (range: 36–70 years). The radiographic parameters, including the radial length, the radial inclination angle, the ulnar variance, and the volar tilt, as well as clinical outcomes, including wrist and forearm range of motion (ROM), grip strength, the Mayo Modified Wrist Score (MMWS), and the disabilities of the Arm, Shoulder, and Hand (DASH) score, were examined at 3 months and 18 months after operation and compared with the preoperative state. The paired *t*‐test was used for statistical analysis.

**Results:**

After corrective osteotomy combined with volar and dorsal plate fixation, all included patients were followed up for 18 months, and there was no surgical site infection. Patients reported postoperative pain due to the irritation of extensor tendon (two cases) and wrist arthritis (two cases). The radial length increased from 1.34 ± 2.34 mm to 9.25 ± 2.65 mm and 9.03 ± 2.47 mm at 3 months and 18 months postoperatively (*t =* 8.257, 7.954, all *p <* 0.05). The radial inclination angle increased from 6.45° ± 0.76° to 19.35° ± 3.43° and 19.03° ± 3.63° at 3 and 18 months (*t =* 12.517, 12.122, all *p <* 0.05). The ulnar variance decreased from 5.11 ± 0.23 mm to 1.32 ± 0.31 mm and 1.54 ± 0.62 mm at 3 and 18 months (*t =* 4.214, 4.895, all *p <* 0.05). The volar tilt was corrected from 4.47° ± 3.46° to 15.51° ± 2.72° and 14.12° ± 2.41°, respectively (*t* = 11.247, 10.432, all *p <* 0.05). Moreover, wrist ROM increased from 42.53° ± 8.99° to 98.70° ± 7.61° and 101.24° ± 7.66° (*t* = 41.433, 46.627, all *p <* 0.05), while forearm ROM was increased from 94.82° ± 6.54° to 134.47° ± 5.06° and 137.24° ± 5.52°, respectively (*t* = 31.507, 32.584, all *p <* 0.05). Similarly, grip strength, MMWS, and DASH were also remarkably improved. There were no significant differences in the wrist and forearm ROM, grip strength, MMWS, and DASH scores between follow‐up at 3 and 18 months (all *p* > 0.05).

**Conclusions:**

Corrective osteotomy with volar and dorsal fixation can improve recovery of volar tilt, relieve wrist pain, restore wrist and forearm function, and increase grip strength of patients with malunion of intra‐articular fractures of the distal radius.

## Graphical abstract

This retrospective study evaluates the clinical value of corrective osteotomy combined with volar and dorsal plate fixation for the treatment of malunion of intra‐articular fractures of the distal radius.

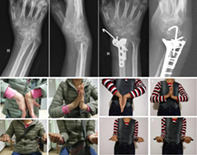

## Introduction

Malunited distal radius fracture is a common clinical injury characterized by restricted movement and rotation of the forearm and impaired handgrip that can elicit considerable wrist pain and disability^1^, ^2^, ^3^. Improper management of malunion of intra‐articular fracture could accelerate the development of arthritis and seriously affect patient quality of life^4^. The main causes of malunited distal radius fracture include re‐displacement after closed reduction of the distal radius fracture and lack of timely treatment of distal radius fracture in the early stage^5^. Therefore, it is of great significance to restore the normal anatomical structure and mechanical properties of the distal radius as soon as possible to relieve pain and improve wrist function^6^, ^7^.

An increasing body of evidence suggests that corrective osteotomy with volar fixation is an effective method for treating distal radius fracture malunion due to its unique advantages, accounting for its increasing use by orthopaedists^2^, ^8^, ^9^. Ingrid Andreasson *et al*. reported good long‐term follow‐up outcomes for 37 patients with malunited fractures of distal radius who underwent corrective osteotomy using a volar plate^10^. Similarly, Kunihiro *et al*. reported that volar fixation had lower surgical technical requirements and fewer complications than dorsal fixation^11^. However, compared with corrective osteotomy with dorsal fixation, osteotomy with volar fixation is often unable to completely correct volar tilt deformities, leading to wrist instability, limiting wrist movement, and even causing joint pain^11^. Martinez‐Mendez *et al*. speculated that insufficient volar tilt correction might result from the poor healing of dorsal bone or insufficient screw fixation in poor bone quality^12^. Indeed, no consensus has been reached on whether dorsal fixation combined with volar fixation may improve the recovery of volar tilt^13^.

In this study, 17 cases of malunion of intra‐articular fracture of the distal radius treated by corrective osteotomy with volar and dorsal plate fixation were retrospectively analyzed. And the aims of the retrospective study were to (i) evaluate the clinical therapeutic effect of corrective osteotomy combined with volar and dorsal plate fixation in patients with malunion of intra‐articular fractures of the distal radius; (ii) provide a comprehensive overview of complications of this type of osteotomy and analyze the possible reasons; (iii) provide clinicians with an alternative surgical approach for malunion of intra‐articular fractures of the distal radius.

## Patients and Methods

### Study Design and Participants

Patients who met the following inclusion criteria were included in this study: (i) limited flexion and extension of the wrist joint; (ii) obvious deformity and pain, restricted forearm rotation, impaired grip strength; (iii) radiographic parameters of malunion: the displacement of the main fracture mass on the articular surface of the wrist joint >2 mm, shortening of distal radius >5 mm, volar tilt >15° or < 10°; (iv) shift of dorsal fracture and abnormal position of malunion, which cannot be reversed with single volar fixation. Patients who met the following criteria were excluded: (i) pathological fracture; (ii) fracture with symptoms of the median nerve and ulnar nerve injury; and (iii) patients with severe cardiopulmonary complications, unable to tolerate surgery. This study was approved by the Ethics Committee of Liuzhou Traditional Chinese Medical Hospital (2016JAN‐KY‐YN‐005‐01) and was conducted at the Liuzhou Traditional Chinese Medical Hospital. All operations were carried out in accordance with the ethical standards in the 1964 Declaration of Helsinki.

### Operative Procedures

#### Anesthesia, Approach, and Exposure

All operations were performed by the same surgical team. After anesthesia, the patients were placed supine with the upper limb abducted on the operating table. A tourniquet was attached to the upper extremity before surgery. A modified Henry incision (about 8 cm in length) was made on the distal forearm. The radial flexor carpi and flexor pollicis longus were moved aside. An L‐shaped incision was made on the anterior rotator muscle to expose the malunited fracture site. A straight incision (about 5 cm in length) was made at the distal end of the dorsal wrist of the forearm (radial Lister). A longitudinal incision was made between the 2nd and 3rd extensor tendon sheath. The extensor retinaculum was cut off, and the extensor pollicis longus tendon was pulled to the radial side to expose the fracture and articular surface.

#### Reduction and Fixation

The initial fracture was evaluated based on preoperative X‐ray and CT of the carpal joints. The periosteal callus was carefully separated using an osteotome. After determining the original fracture line, a volar osteotomy was carried out first along the original fracture line and a dorsal osteotomy along the original fracture line. After two osteotomies were conducted to separate the fracture, traction and reposition of the fracture were carried out.

Kirschner wires were used to fix the fractures after the radial inclination, radial length, and volar tilt was restored. The fractures were then fixed with a T‐shaped volar anatomical locking plate (Kehui, China) while avoiding screw penetration through the dorsal cortex. Six of the 17 patients received autogenous bone graft implantation due to large bone defects. A 1.5‐mm mini‐plate (Kehui, China) was placed longitudinally on the ulnar side of Lister's nodules. The distal screw was firmly attached to the subchondral bone of the articular surface of the radius for fixation, and the dorsal screw was fixed with a single cortex.

### Postoperative Management

After the operation, all patients received prophylactic antibiotics for 24 h. Two of the 17 patients had unstable fractures, fixed externally with a wrist brace for 2 weeks. Rehabilitation was mainly adopted to train the grip strength of fingers in the 1st week postoperatively, followed by wrist flexion and extension exercises in the 2nd week. The forearm rotation function exercise was started in the 3rd week. Six weeks postoperatively, strength training began, and training of activities of daily living gradually began.

### Observation Indicators

Follow‐up was conducted on an outpatient basis as patients with an X‐ray examination and assessment of the functional performance of the wrist joint. The recovery outcomes of wrist function were assessed by radiological and clinical measurements. Radiographic parameters included radial inclination, radial length, volar tilt, and ulnar variance, while clinical parameters included wrist and forearm ROM, grip strength, MMWS, DASH, which were determined as previously described^14^, ^15^.

#### Radial Inclination

The radial inclination is a measurement made on the anteroposterior (AP) projection of the wrist as an angle of the distal radial surface concerning a line perpendicular to the shaft, with a normal range of about 21°–25°. It is one of the important indicators of distal radius fracture reduction.

#### Radial Length

Radial length (also known as radial height) is the distance between two lines drawn perpendicular to the long axis of the radius on the AP projection from the apex of the radial styloid and the level of the ulnar aspect of the articular surface. It is used to evaluate the shortening of the radius because of impaction or displacement.

#### Volar Tilt

Volar tilt is a measurement made on the lateral projection of the wrist as an angle of the distal radial surface with respect to a line perpendicular to the shaft, with a normal range of about 10°–15°. An angle >15° indicates dorsal insertion segment instability. Angle reduction is indicative of dorsal angulation.

#### Ulnar Variance

A normal or neutral ulnar variance means the surface of the distal ulna and radius are equal. A negative variance occurs when the ulna is shorter than the radius, while a positive ulnar variance describes an ulna longer than the radius.

#### Wrist Range of Motion

A goniometer was used to measure wrist extension and wrist flexion. The wrist extension and flexion value usually represent the wrist range of motion.

#### Forearm Rotation Range of Motion

The patient was asked to bend the elbow and hold a pen with both hands, with the palm gradually ahead and backward until reaching the end range of motion. A goniometer was used to measure the trajectory of the pen, which represented the forearm rotation range of motion.

#### Grip Strength

Grip strength was the percentage of the contralateral limb^8^. A hydraulic grip dynamometer was used to measure the grip strength, which evaluated the recovery of wrist function.

#### The Mayo Modified Wrist Score (MMWS)

The Modified Mayo Wrist Score consists mainly of the doctor's assessment of pain, active flexion and extension, grip strength, and the ability to return to normal work or activity, each worth 25 points, for a total of 100 points. A score of 90–100 was excellent, 80–90 was good, 65–79 was acceptable, and 0–64 was poor^16^.

#### The Disabilities of the Arm, Shoulder, and Hand (DASH)

The Disabilities of the Arm, Shoulder, and Hand questionnaire (DASH) contains a 30‐item disability/symptom scale, ranging from 0 to 100, with higher scores indicating worse upper‐extremity function^16^, ^17^.

### Statistical Analysis

All numerical data were presented as mean ± SD. SPSS statistical software package (SPSS 19.0 version; SPSS Inc., Chicago, Illinois, USA) was used for statistical analysis. The paired *t*‐test was used to evaluate differences between the preoperative state and the postoperative state at 3 and 18 months. A *p*‐value less than 0.05 was statistically significant.

## Results

### General Results

**Fig. 1:**
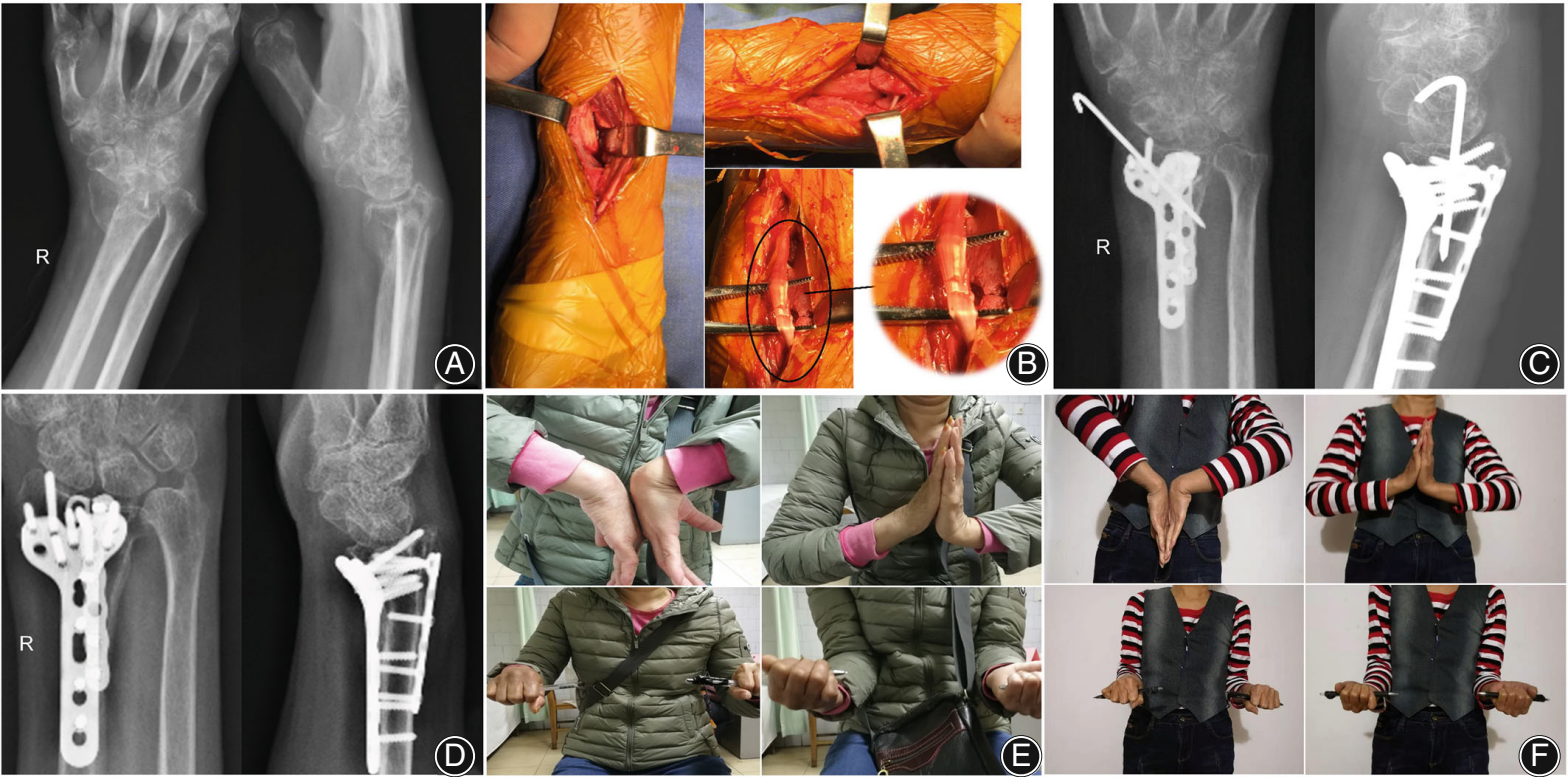
A 58‐year‐old woman suffered from malunion of distal radius fractures and a shortened radius. (A) Anteroposterior and lateral radiographs of the right radiocarpal joint. (B) Corrective osteotomy was performed along the original fracture line from the volar and dorsal approaches. The extensor pollicis longus tendon was inserted into the fracture site, and a part of it was damaged. (C) Corrective osteotomy with volar and dorsal plate fixation restored radius length and volar tilt. Anteroposterior and lateral radiographs at 3 months postoperatively. (D) Anteroposterior and lateral radiographs of a slightly shorter radius length at 18 months postoperatively. (E) Wrist function was evaluated by flexion and extension of wrist and pronation and supination of the forearm at 3 and (F) 18 months postoperatively

**Fig. 2:**
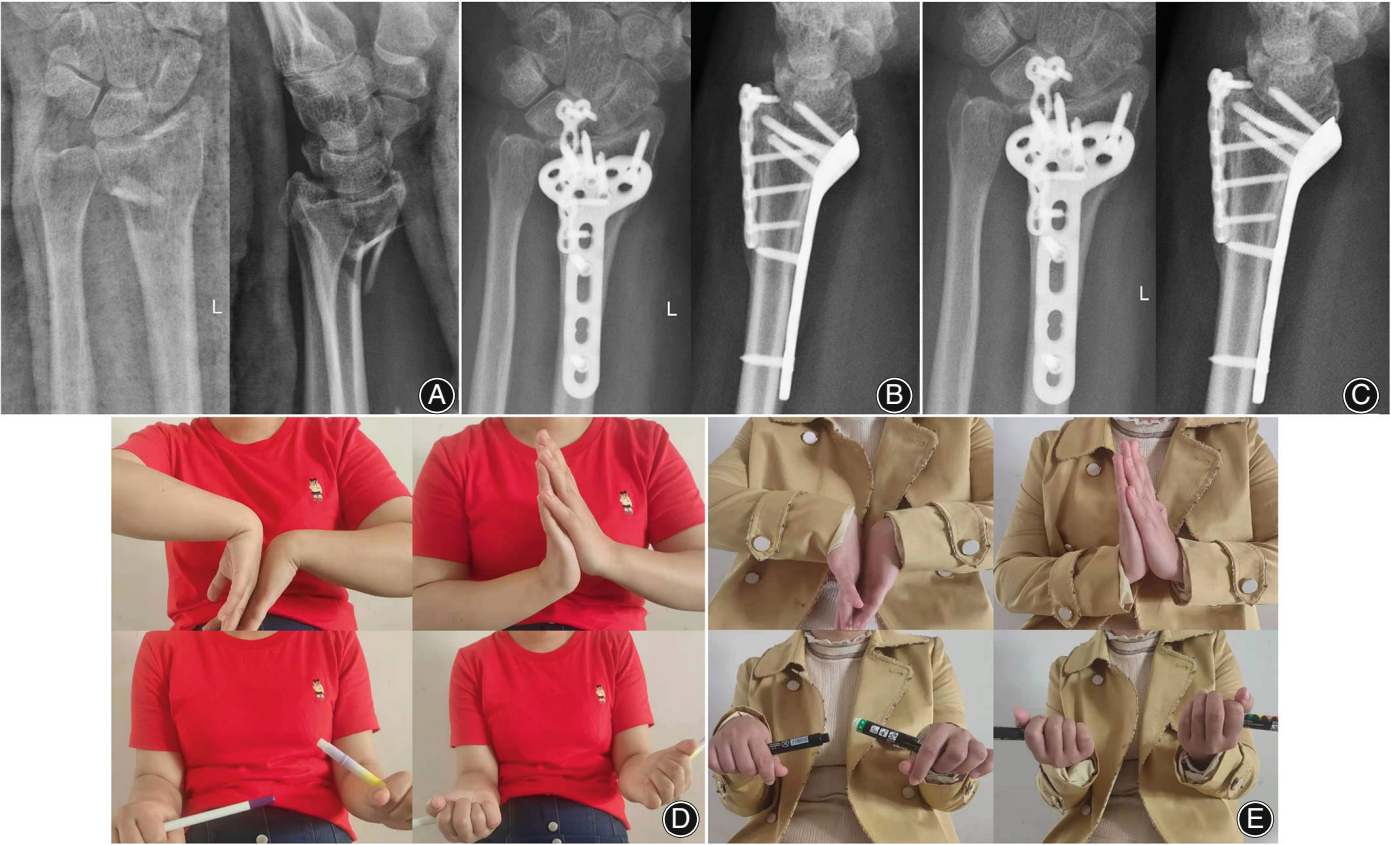
A 45‐year‐old woman suffered from malunion of distal radius fractures, with obvious fracture displacement and the loss of volar tilt. (A) Anteroposterior and lateral radiographs of the left radiocarpal joint. (B) Corrective osteotomy with volar and dorsal plate fixation restored volar tilt at 3 months postoperatively with anteroposterior and lateral radiographs shown. (C) Anteroposterior and lateral radiographs of the radius length and volar tilt within the normal range at 18 months postoperatively. (D) Wrist function was evaluated by flexion and extension of wrist and pronation and supination of the forearm at 3 and (E) 18 months postoperatively

**Fig. 3:**
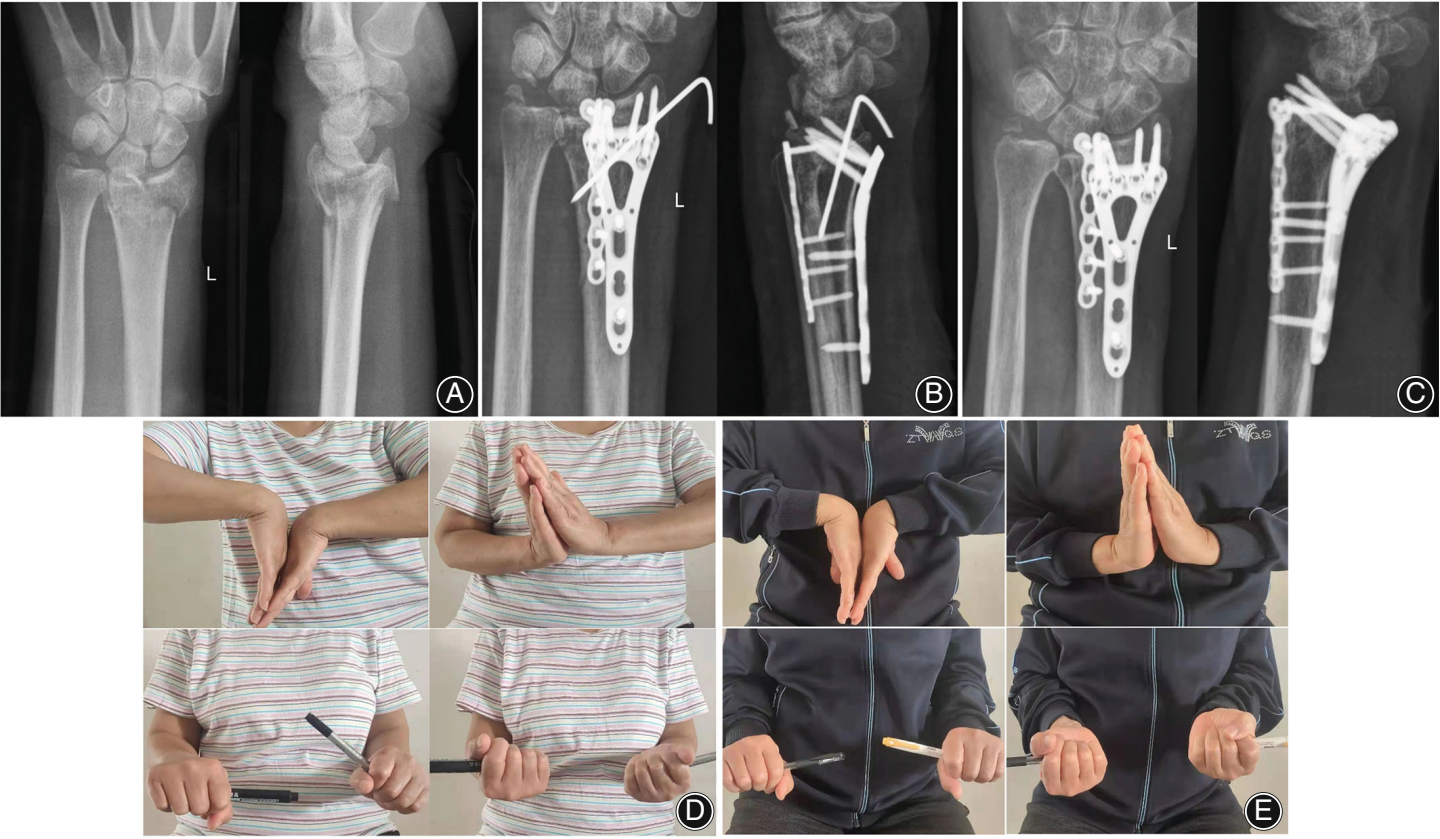
A 70‐year‐old woman suffered from malunion of distal radius fractures, radius shortening, and the loss of volar tilt. (A) Anteroposterior and lateral radiographs of the left radiocarpal joint. (B) Anteroposterior and lateral radiographs of the left radiocarpal joint at 3 months show restoration of the radius height and volar tilt. (C) Anteroposterior and lateral radiographs of the radius length and volar tilt within the normal range at 18 months. (D) Wrist function was evaluated by flexion and extension of wrist and pronation and supination of the forearm at 3 and (E) 18 months postoperatively

The detailed demographic information for all 17 included patients followed‐up for 18 months is provided in Table S1. No surgical site infection and rejection of allograft were observed. Two patients suffered from postoperative pain due to the irritation of the extensor tendon, and the pain symptoms were relieved after fracture healing and steel plate removal. Another two patients suffered from wrist arthritis. The mean fracture healing time was 4 months (range: 3 to 6 months). All included patients were followed up by outpatient clinic visits. Three typical cases are shown in Figures 1, 2, 3.

### Radiographic Analysis

To explore the clinical value of corrective osteotomy with volar and dorsal fixation on malunion of intra‐articular fractures of the distal radius, we first analyzed the changes in radiographic parameters of the wrist joint. We found that the radial length increased from 1.34 ± 2.34 mm (preoperatively) to 9.25 ± 2.65 mm and 9.03 ± 2.47 mm at 3 and 18 months postoperatively (*t* = 8.257, 7.954, all *p* < 0.05). The radial inclination angle was increased from 6.45° ± 0.76° to 19.35° ± 3.43° and 19.03° ± 3.63° at 3 and 18 months (*t* = 12.517, 12.122, all *p* < 0.05), respectively. The ulnar variance decreased from 5.11 ± 0.23 mm to 1.32 ± 0.31 mm and 1.54 ± 0.62 mm at 3 and 18 months (*t* = 4.214, 4.895, all *p* < 0.05). While the volar tilt was corrected from 4.47° ± 3.46° to 15.51° ± 2.72° and 14.12° ± 2.41° (*t* = 11.247, 10.432, all *p* < 0.05), respectively. In addition, the radial length in two patients was slightly reduced to about 10 mm at 18 months post‐operation, although both patients were satisfied with the degree of recovery of wrist function. There were no significant differences in radial length, radial inclination, volar tilt, and ulnar variance between 3 and 18 months postoperatively (all *p >* 0.05) (Table 1). These data suggested that corrective osteotomy using volar and dorsal fixation significantly improved the radial length, radial inclination, volar tilt, and ulnar variance of patients with malunion of intra‐articular fractures of the distal radius.

**TABLE 1:** Clinical and radiometric parameters before and after surgery (Mean ± SD)

Parameter	Before surgery	3 months after surgery	18 months after surgery	*t*	*P* value
Wrist ROM,°	42.53 ± 8.99	98.70 ± 7.61	101.24 ± 7.66	41.433 46.627 2.125	<0.001^†^ < 0.001^¶^ 0.36^∆^
Forearm ROM,°	94.82 ± 6.54	134.47 ± 5.06	137.24 ± 5.52	31.507 32.584 1.761	< 0.001^†^ 0.002^¶^ 0.52^∆^
Grip strength, %^€^	46.12 ± 4.90	77.59 ± 4.78	79.29 ± 4.83	27.614 29.470 1.034	< 0.001^†^ < 0.001^¶^ 0.31^∆^
Radial inclination,°	6.45 ± 0.76	19.35 ± 3.43	19.03 ± 3.63	12.517 12.122 0.451	0.002^†^ < 0.001^¶^ 0.41^∆^
Radial length, mm	1.34 ± 2.34	9.25 ± 2.65	9.03 ± 2.47	8.275 7.954 0.315	0.003^†^ < 0.001^¶^ 0.46^∆^
Volar tilt,°	4.47 ± 3.46	15.51 ± 2.72	14.12 ± 2.41	11.247 10.432 1.125	< 0.001^†^ < 0.001^¶^ 0.38^∆^
Ulnar variance, mm	5.11 ± 0.23	1.32 ± 0.31	1.54 ± 0.62	4.214 4.895 1.004	< 0.001^†^ < 0.001^¶^ 0.61^∆^
MMWS	65.82 ± 6.89	82.82 ± 5.63	83.59 ± 4.94	20.554 21.790 0.421	< 0.001^†^ < 0.001^¶^ 0.32^∆^
DASH	53.76 ± 6.76	37.23 ± 4.48	35.71 ± 3.93	19.460 17.000 1.058	< 0.001^†^ < 0.001^¶^ 0.29^∆^

^†^, stands for 3 months post‐operation *vs* pre‐operation; ^¶^, stands for 18 months post‐operation *vs* pre‐operation; ^∆^, stands for 3 months post‐operation *vs* 18 months post‐operation; ^€^, percentage of the contralateral limb.

### Clinical Evaluation

To further investigate the long‐term functional outcomes of this approach, the ROM of the wrist and forearm, and grip strength were determined. The results showed that both the wrist and forearm ROM were significantly improved after 3 months (wrist ROM 42.53° ± 8.99° *vs* 98.70° ± 7.61°, forearm ROM 94.82° ± 6.54° *vs* 134.47° ± 5.06°) and 18 months (wrist ROM 42.53° ± 8.99° *vs* 101.24° ± 7.66°, forearm ROM 94.82° ± 6.54° *vs* 137.24° ± 5.52°). Moreover, the wrist ROM and forearm ROM observed at 3 and 18 months postoperatively were comparable (137.24° ± 5.52° and 101.24° ± 7.66°, respectively). In addition, the grip strength increased from 46.12 ± 4.90 to 77.59 ± 4.78 (at 3 months) (*t* = 27.614, *p* < 0.05), and 79.29 ± 4.83 (at 18 months) (*t* = 29.470, *p* < 0.05) (Table 1). These findings suggested that corrective osteotomy using volar and dorsal fixation significantly improved wrist and forearm ROM and grip strength.

### Functional Scores

The therapeutic effects of this approach were subsequently determined by the MMWS and DASH scores. The MMWS analysis showed that the value of MMWS were respectively 82.82 ± 5.63 and 83.59 ± 4.94 at 3 months and 18 months post‐operation, which were significantly higher than those before operation (65.82 ± 6.89) (*t* = 20.554, 21.790, all *p* < 0.05). Moreover, the DASH scores declined from 53.76 ± 6.76 (preoperative results) to 37.23 ± 4.48 and 35.71 ± 3.93 at 3 months and 18 months post‐operation, respectively (*t* = 19.460, 17.000, all *p* < 0.05). There were no significant differences in MMWS and DASH values between 3 and 18 months postoperatively (*t* = 0.421, 1.058, all *p* > 0.05) (Table 1). These results suggested that corrective osteotomy using volar and dorsal fixation can maintain MMWS and DASH scores stably at 18 months.

### Complications

No surgical site infection and rejection of allograft were observed. Two patients suffered from postoperative pain due to the irritation of the extensor tendon 5 months after the operation, and the pain symptoms were relieved after fracture healing and steel plate removal. In addition, two patients suffered from wrist arthritis 6 months after the operation, but significant improvement was achieved after rehabilitation. We speculated that the latter complication was attributed to the severity of the injury, as both patients were diagnosed with a “C” fracture.

## Discussion

In the present study, we found that corrective osteotomy with volar and dorsal fixation could conspicuously correct the deformities of volar tilt and provide greater stability to the wrist joint, which can effectively relieve wrist pain and improve the wrist and forearm function, and increase grip strength, leading to good long‐term functional outcomes. This approach represents a potential therapeutic strategy for malunion of intra‐articular fracture of the distal radius.

### Advantages and Disadvantages of Volar Fixation and Dorsal Fixation

Numerous clinical studies have demonstrated that compared with dorsal fixation, using a volar locking plate for corrective osteotomy leads to fewer complications, which was increasingly welcomed by orthopaedic surgeons^7^, ^18^, ^19^, ^20^. Tarallo *et al*. reported that 14 out of 20 patients with malunion of intra‐articular fracture of the distal radius treated by corrective osteotomy with a volar locking plate exhibited excellent results, and six cases achieved good results^21^. However, a comparative study of corrective osteotomies with dorsal fixation and corrective osteotomies with volar fixation showed that volar fixation was insufficient in completely correcting volar tilt, an important radiographic parameter for obtaining long‐term good functional outcomes^22^. Moreover, volar fixation alone made it easier for the tip of the screw to penetrate the dorsal cortex, resulting in the extensor tendon injury^7^, ^23^. In addition, its application may increase the incidence of extensor tendon irritation in dorsally displaced radial fractures^24^. Moreover, Kunihiro *et al*. noted that the volar fixation had a significantly greater volar tilt under correction for −9.4° than did the dorsal fixation for −1.2°^11^. It has been reported that intra‐articular correction is more convenient through the dorsal approach^25^, and the articular surface can be observed directly to deal with dorsal fragments or irreversible depressions^26^.

### Advantages of the Combination of Dorsal and Volar Fixation for Malunion of Intra‐Articular Fracture of the Distal Radius

In our study, the correction of volar tilt by combining dorsal and volar fixation was 11.04° and 9.65° at 3 and 18 months postoperatively, respectively. These findings suggested that dorsal fixation may assist in correcting volar tilt by volar fixation. In addition, the increase of radius length after the operation was stably maintained at 3 and 18 months post‐operation. Good radiographic parameters had positive effects on the functional outcomes. After corrective osteotomy with volar and dorsal fixation, the MMWS was improved from 65.82 ± 6.89 to 82.82 ± 5.63 at 3 months, while the DASH score decreased from 53.76 ± 6.76 to 37.23 ± 4.48. Importantly, these parameters remained stable at 18 months, indicating this approach yielded an effective and sustained effect in restoring patient wrist function.

### Preventive Measures of Extensor Tendon Irritation

Oka *et al*. reported that six out of nine patients experienced tendon problems after corrective osteotomy combined with dorsal fixation^13^. Our study found that two out of 17 patients experienced postoperative pain due to extensor tendon irritation, which indicated that this approach might have fewer side effects, which may be attributed to the following reasons. First, we restored the bone fragments on the dorsal surface, which may have interfered with the extensor tendons. Moreover, it was more objective to identify whether extensor tendon was damaged. Besides, the volar screws could be directly observed during the operation to avoid piercing the dorsal cortex. In addition, a 1.5‐mm mini‐plate was placed longitudinally on the ulnar side of Lister's nodules, which was extremely thin to reduce the interference to the extensor tendon. Last but not least, deformation of the screw cap caused by excessive force was avoided when tightening screws, and keeping the screw cap smooth was pivotal to reducing irritation to the extensor tendon.

### Bone Grafting

In addition, no consensus has been reached on the necessity of bone grafting for patients with malunited distal radius fractures. Mathew *et al*. reported that bone grafting was indispensable for patients with insufficient bone mass and poor healing ability^18^. However, Ozer *et al*. pointed out no significant clinical or statistical difference in time to union and outcomes between the bone grafts and non‐bone grafts^27^. Consistent with Mugnai *et al*.’s findings^19^, our data suggested that bone grafting was not necessary for every patient but exhibited significant value for patients with large bone defects.

### Limitation

Although our study provided compelling evidence of the therapeutic effect of corrective osteotomy with a volar and dorsal fixation on malunion of intra‐articular fractures of the distal radius, several limitations should be considered. First, this was a retrospective study with limited sample size. Accordingly, large‐scale multicenter prospective case studies are warranted to confirm the advantages of this approach in malunion of intra‐articular fractures of the distal radius. Besides, our results may have been affected to a certain extent by the loss of follow‐up data since postoperative radiographs at 6 and 12 months were not available in some cases. However, radiographic parameters and functional outcomes at 3 and 18 months substantiated the therapeutic effects of our approach. Furthermore, the duration of our study was not long enough. A longer follow‐up duration is still required to identify the therapeutic effects of corrective osteotomy with a volar and dorsal fixation on functional outcomes.

### Conclusions

In summary, corrective osteotomy with volar and dorsal fixation can effectively correct volar tilt, reduce wrist pain, and improve wrist and forearm function and grip strength. This approach is effective for treating malunion of intra‐articular fractures of the distal radius. Indeed, multicenter studies with a larger study population are warranted to corroborate our findings.

## GRANT SOURCES

This study was financially supported by National Natural Science Foundation of China (No. 82174140, 82174401, 82104164, 81804121, 81973870 and 81973881), Postdoctoral Research Foundation of China (No.: 2018M632154), Natural Science Foundation of Zhejiang Province (No. LY22H270003, LY19H270006 and LQ19H080001), the Joint Funds of the Zhejiang Provincial Natural Science Foundation of China (under Grant No. LBY22H270008), Traditional Chinese Medical Administration of Zhejiang Province (No. 2022ZX005, 2022ZB119 and 2021ZB090), Zhejiang Medical and Health Science and Technology Project (No. 2021KY222), Zhejiang Chinese Medical University Scientific Research Fund Project (No. 2021JKZDZC02, 2021JKZKTS036A, 2021JKJNTZ022B), General Research Project of Zhejiang Provincial Education Department “Special Project for the Reform of Cultivation Mode of Professional Degree Graduate Students in Higher Education Institutions” (No. Y202145932), Postgraduate Science Research Fund of Zhejiang Chinese Medical University (No. 2021YKJ02, 2020YKJ07).

## DISCLOSURE

All named authors have no conflicts of interest to disclose in relation to this article.

## Supporting information


**Table S1** Detailed demographic information of 17 included patients.
