# Supplementary material for: Corrective Osteotomy with Volar and Dorsal Fixation for Malunion of Intra‐Articular Fracture of the Distal Radius: A Retrospective Study
Source: Orthop Surg. 2022 Jul 22;14(8):1751–8. doi: 10.1111/os.13409 (PMC9363726; doi:10.1111/os.13409)
Supplement: Supplementary file 1 — Table S1 Detailed demographic information of 17 included patients. [file OS-14-1751-s001.docx]

| **Supplementary Table S1**  **Detailed demographic information of 17 included patients.** | | | | | | | | |
| --- | --- | --- | --- | --- | --- | --- | --- | --- |
| **No.** | **Surgery time** | **Sex** | **Age^*^** | **Location** | **Fracture**  **classification^†^** | **Occupation** | **Mechanism of injury** | **Time from injury to surgery (m^#^)** |
| **1** | Jan/2016 | F | 45 | Right wrist | B1-2 | Laborer | Low energy | 2 |
| **2** | May/2016 | F | 56 | Right wrist | C3-2 | Laborer | High energy | 3 |
| **3** | Jul/2016 | M | 36 | Left wrist | B1-2 | Official | High energy | 1 |
| **4** | Dec/2016 | F | 50 | Right wrist | C2-3 | Laborer | High energy | 2 |
| **5** | Feb/2017 | M | 61 | Left wrist | C3-2 | Retired | Low energy | 2.3 |
| **6** | Jul/2017 | F | 65 | Right wrist | B2-2 | Retired | Low energy | 3 |
| **7** | Sep/2017 | F | 58 | Right wrist | C3-3 | No data | Low energy | 2 |
| **8** | Oct/2017 | M | 56 | Left wrist | B1-2 | Laborer | Low energy | 1.6 |
| **9** | Nov/2017 | F | 70 | Left wrist | C2-3 | Retired | Low energy | 2 |
| **10** | Jan/2018 | M | 53 | Right wrist | C3-3 | Laborer | High energy | 3 |
| **11** | Jan/2018 | M | 55 | Left wrist | B2-1 | Official | High energy | 2.1 |
| **12** | Feb/2018 | F | 58 | Right wrist | B2-2 | Laborer | Low energy | 2.5 |
| **13** | Jun/2018 | F | 64 | Left wrist | B2-1 | Retired | Low energy | 3 |
| **14** | Jun/2018 | F | 50 | Right wrist | B2-3 | No data | High energy | 4 |
| **15** | Jul/2018 | M | 55 | Left wrist | C3-2 | Official | High energy | 1.3 |
| **16** | Sep/2018 | M | 58 | Right wrist | C3-2 | No data | High energy | 3.2 |
| **17** | Nov/2018 | F | 45 | Left wrist | C1-3 | No data | High energy | 1 |
| Note. **^*^**, year; ^†^, AO/ASIF classification; **^#^**, month. | | | | | | | | |
